# Colossal oxygen vacancy formation at a fluorite-bixbyite interface

**DOI:** 10.1038/s41467-020-15153-8

**Published:** 2020-03-13

**Authors:** Dongkyu Lee, Xiang Gao, Lixin Sun, Youngseok Jee, Jonathan Poplawsky, Thomas O. Farmer, Lisha Fan, Er-Jia Guo, Qiyang Lu, William T. Heller, Yongseong Choi, Daniel Haskel, Michael R. Fitzsimmons, Matthew F. Chisholm, Kevin Huang, Bilge Yildiz, Ho Nyung Lee

**Affiliations:** 1 Oak Ridge National Laboratory, Oak Ridge, TN 37831 USA; 2 Laboratory for Electrochemical Interface, Department of Nuclear Science and Engineering, Massachusetts Institute of Technology, 77 Massachusetts Avenue, 24-210, Cambridge, MA 02139 USA; 3 Department of Mechanical Engineering, University of South Carolina, Columbia, SC 29208 USA; 4 Advanced Photon Source, Argonne National Laboratory, Argonne, IL 60439 USA; 5 Department of Physics and Astronomy, University of Tennessee at Knoxville, Knoxville, TN 37996 USA; 6 Present Address: Department of Mechanical Engineering, University of South Carolina, Columbia, SC 29208 USA; 7 Present Address: Center for High Pressure Science and Technology Advanced Research, Beijing, China

**Keywords:** Electronic devices, Materials for energy and catalysis, Nanoscale materials, Nanoscale materials, Surfaces, interfaces and thin films

## Abstract

Oxygen vacancies in complex oxides are indispensable for information and energy technologies. There are several means to create oxygen vacancies in bulk materials. However, the use of ionic interfaces to create oxygen vacancies has not been fully explored. Herein, we report an oxide nanobrush architecture designed to create high-density interfacial oxygen vacancies. An atomically well-defined (111) heterointerface between the fluorite CeO_2_ and the bixbyite Y_2_O_3_ is found to induce a charge modulation between Y^3+^ and Ce^4+^ ions enabled by the chemical valence mismatch between the two elements. Local structure and chemical analyses, along with theoretical calculations, suggest that more than 10% of oxygen atoms are spontaneously removed without deteriorating the lattice structure. Our fluorite–bixbyite nanobrush provides an excellent platform for the rational design of interfacial oxide architectures to precisely create, control, and transport oxygen vacancies critical for developing ionotronic and memristive devices for advanced energy and neuromorphic computing technologies.

## Abstract (web summary)

Oxygen vacancies can impart interesting properties in complex oxides, but specific architectures designed to create high-density oxygen vacancies are largely unknown. Here the authors report a fluorite-bixbyite nanobrush platform to tune interfacial oxygen and show that an atomically well-defined heterointerface can induce charge modulation.

## Introduction

Owing to the critical role of tunable oxygen defects, mixed ionic and electronic conducting oxides have become key components in many energy technologies, including energy conversion and generation^1^, solar hydrogen production^2^, humidity/gas detection^3^, and catalytic conversion of toxic species in automobile exhausts^4^. In particular, ceria (CeO_2_) has attracted extensive attention for its good ionic conductivity and remarkable redox capability^5^ and hence is widely used as a heterogeneous catalyst^6^ and oxygen storage component^4^. Because the key element is the oxygen vacancy^7^, the creation of oxygen vacancies is a prerequisite for such CeO_2_-based ionic materials and devices. CeO_2_ has a fluorite-type structure with a coordination number of 8 for Ce to O atoms^8^, so aliovalent chemical doping (e.g., yttrium, samarium, and gadolinium) into CeO_2_ is one of the conventional approaches^9^ to create oxygen vacancies. Nanoscaling, interfacing, and straining thin films are also known mechanisms to create oxygen vacancies^10–12^. However, the implementation of these approaches for practical applications requires further advancements because the fundamental understanding of oxygen vacancy formation at the atomic level is still lacking.

It was proposed that a space charge region (SCR)^12–14^ could be formed by interfacing the fluorite CeO_2_ (space group $Fm\bar 3m$, *a* = 5.412 Å) with a bixbyite oxide, such as Y_2_O_3_ (space group $Ia \bar 3$, *a* = 10.607 Å), along the [100] direction^15,16^. The bixbyite structure has a “pseudofluorite” structure with an ordered array of vacant oxygen sites occurring on every fourth site (Supplementary Fig. 1), providing an ideal atomic arrangement for forming an interfacial charge layer that originates from the valence mismatch between Ce(4+) and Y(3+). However, the formation of an atomically well-defined (001) interface between the two binary oxides is energetically unfavorable, as the (001) surface has the highest surface energy among the low-index surfaces for both materials. In contrast, the (111) surfaces of fluorite and bixbyite structures are energetically more favorable because of their low surface energy^17,18^. Thus, determining the viability of the artificial creation of oxygen vacancies at an atomically well-defined (111) interface and understanding the (111) fluorite–bixbyite interface could open a new avenue toward the development of high-performance oxide-based ionotronic devices. Moreover, the Ce^4+^O_2_/Y^3+^_2_O_3_ interface offers an interfacial condition similar to that of semiconductor and perovskite-oxide heterointerfaces having polar discontinuity^19–21^. Therefore, understanding the details of the interfacial oxygen vacancy formation reported herein will provide useful insights into developing a novel concept of two-dimensional (2D) ionic channels using atomically engineered oxide interfaces.

**Fig. 1:**
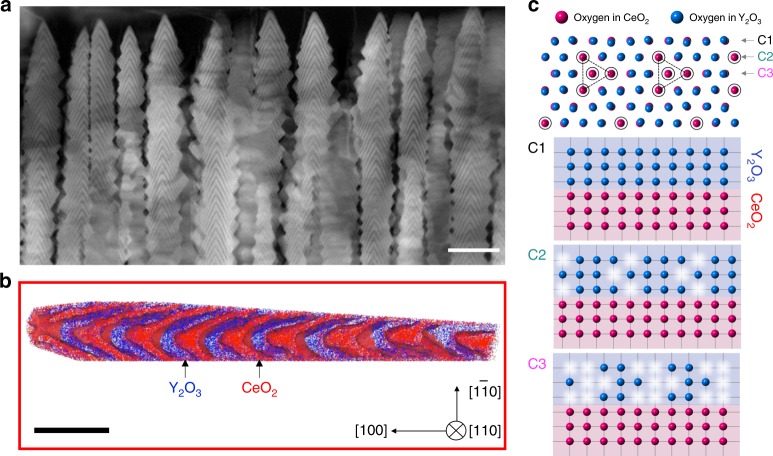
CeO_2_/Y_2_O_3_ nanobrush and the atomic configurations of the (111) interface. **a** A cross-sectional HAADF-STEM image of a [(CeO_2_)_6 u.c._/(Y_2_O_3_)_2 u.c._]_200_ nanobrush superlattice with a thickness of 1.4 μm grown on a (001) YSZ-buffered silicon substrate, taken along the [110] direction. Because of the nonuniform thickness of sublayers originating from faceting, we use the average thickness of the individual layers. Note that oxide nanostructures were grown on (001) YSZ-buffered silicon substrates, as TEM imaging for samples grown directly on YSZ was difficult because of severe charging of electrons. The scale bar corresponds to 50 nm. **b** Atom probe tomography (APT) reconstruction with Ce and Y atoms displayed as red and blue points, respectively, showing a three-dimensional view of the free-standing [(CeO_2_)_6 u.c._/(Y_2_O_3_)_2 u.c._]_200_ internal structure. The scale bar corresponds to 20 nm. **c** Schematic of a (111) interface between CeO_2_ (fluorite) and Y_2_O_3_ (bixbyite), in which red and blue circles represent oxygen atoms in CeO_2_ and Y_2_O_3_, respectively. Cations are not shown. The (111) Y_2_O_3_/CeO_2_ interface is a combination of three different anion arrays, C1, C2, and C3. A cross-section of the C1 array shows a perfect match between numbers of oxygen atoms on interfacing CeO_2_ and Y_2_O_3_ sides. In contrast, on the Y_2_O_3_ side of the interface, a cross-section shows on average one oxygen vacancy every four sites in the C2 array and one oxygen vacancy every two sites in the C3 array.

In this work, we investigate the viability of colossal formation of oxygen vacancies in CeO_2_/Y_2_O_3_ superlattices with atomically well-defined (111) interfaces formed within a nanobrush architecture (Fig. 1). The nanobrush superlattices offer many advantages over 2D thin films, including much larger surface areas and a larger number of interfaces than exist in planar films, which are useful for many applications in various technologically relevant areas^22^.

## Results

### Precision synthesis of a (111) fluorite–bixbyite nanobrush

Figure 1a shows a cross-sectional high-angle annular dark-field (HAADF) image obtained using scanning transmission electron microscopy (STEM) from a 1.4-μm-thick CeO_2_/Y_2_O_3_ nanobrush superlattice grown on a Y_2_O_3_-stabilized ZrO_2_ (YSZ)-buffered (001) silicon substrate. The *Z*-contrast (*Z* denotes the atomic number) HAADF image clearly reveals alternating stacks of CeO_2_ (*Z*_Ce_ = 58) and Y_2_O_3_ (*Z*_Y_ = 39) layers. The [(CeO_2_)_6 u.c._/(Y_2_O_3_)_2 u.c._]_200_ nanobrush superlattice was grown by pulsed laser deposition under an extremely nonequilibrium supersaturated condition, which is far away from the conventional condition optimized for the layer-by-layer growth of 2D thin films. Despite the porous architecture with single-crystalline bristles and the large thickness, the created nanostructure was highly epitaxial and (001)-oriented, as confirmed by X-ray diffraction (XRD) 2*θ*−*θ* scans and pole figure measurements (Supplementary Fig. 2). Based upon the XRD data, the epitaxial orientation relationship of the CeO_2_/Y_2_O_3_ nanobrush superlattice on the YSZ-buffered (001) silicon substrate was Y_2_O_3_(001)∥CeO_2_(001)∥YSZ(001)∥Si(001), a finding also confirmed by selected area electron diffraction (Supplementary Fig. 3). While a variety of oxide nanostructures—such as nanorods^23,24^, nanowires^25^, nanobelts^26^, and nanonails^27^—have been successfully demonstrated, our nanobrush architecture is a revolutionary new form of oxide nanostructure in which the growth is governed by both diffusion-limited aggregation and the shadow effect (Supplementary Fig. 4). The details of the growth mechanism for oxide nanobrush architectures were reported elsewhere^28^. Interestingly, a characteristic chevron pattern can be seen within each bristle. This chevron pattern originates from the {111} facet formation for both CeO_2_ and Y_2_O_3_, owing to the lowest surface energy^17,18^. The interface connection of the {111} facets of the CeO_2_ and Y_2_O_3_ sublayers was the key to the spontaneous formation of oxygen vacancies on the CeO_2_ side, as discussed below.

We performed atom probe tomography (APT) on the CeO_2_–Y_2_O_3_ nanobrush to reveal the three-dimensional (3D) geometry of the nanobristles. Owing to the small nanobristle size (only ~30 nm in the lateral dimension), the growth of a nanobristle on a micron-wide silicon-tip for APT was highly challenging. Nevertheless, we successfully grew a nanobrush sample on a special silicon microtip array and prepared an APT specimen via focused ion beam (FIB) milling, as shown in Supplementary Fig. 5. Figure 1b shows an APT image (see also Supplementary Movie 1 for 3D view) of a (CeO_2_)_6 u.c._/(Y_2_O_3_)_2 u.c._ nanobristle. The APT investigation confirmed the chevron pattern on the nanobristles. The overall “Christmas tree-like” shape of the bristles and the chevron pattern on each bristle are only visible along the <110> direction, whereas the <100> directional view reveals a horizontal layer-by-layer stacking of superlattices, clearly visualizing the 3D geometry of the nanobrush samples.

Figure 1c schematically shows top and cross-sectional views of the oxygen coordination mismatch at the (111) interface between the fluorite and the bixbyite, depicting the underlying mechanism for the formation of oxygen vacancies. Only oxygen atoms are shown to reveal the difference in the oxygen network between (111)CeO_2_ and (111)Y_2_O_3_. While the structure of a (001) fluorite/bixbyite interface is rather simple (Supplementary Fig. 1), the oxygen network along the (111) plane is rather complex, forming a triangular network of oxygen vacant sites in Y_2_O_3_. The cross-sectional views cut along the C1, C2, and C3 directions clearly visualize the oxygen mismatch between the two materials. This interfacial oxygen coordination mismatch can explain the underlying mechanism behind the formation of the interfacial oxygen vacancies. To cope with the valence mismatch in Y^3+^ vs. Ce^4+^, the following two scenarios were considered: (1) formation of an oxygen interstitial in Y_2_O_3_ and (2) formation of an oxygen vacancy in CeO_2_.

### Artificial formation of oxygen vacancies at the (111) interface

**Fig. 2:**
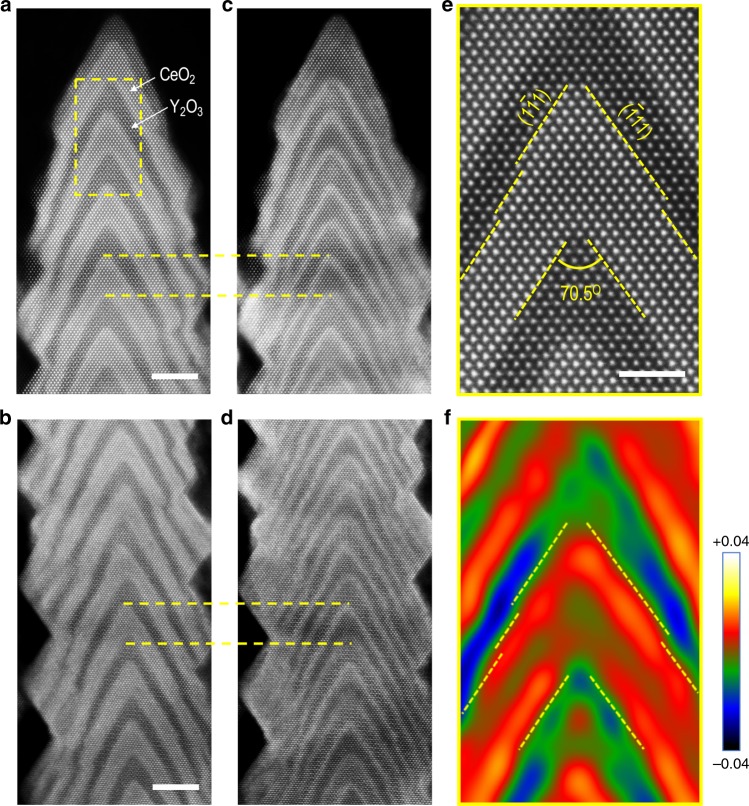
(111) interface and local strain in a free-standing CeO_2_/Y_2_O_3_ nanobristle. **a**, **b** HAADF images and **c**, **d** their corresponding LAADF images taken from the top and middle regions of a free-standing CeO_2_/Y_2_O_3_ nanobristle along the [110] direction. As indicated by the yellow dashed lines, the brighter contrast at the outer parts of the CeO_2_ sublayers in the LAADF images suggests locally enhanced lattice distortions due to the formation of oxygen vacancies. The scale bar corresponds to 5 nm. **e** Magnified HAADF image showing atomically abrupt interfaces and {111} Ce facets between CeO_2_ and Y_2_O_3_ layers. The scale bar corresponds to 2 nm. **f** Geometric phase analysis of the HAADF image shown in **e** reveals a lattice expansion of the outer parts of the CeO_2_ layers.

To confirm the viability of highly confined formation of oxygen vacancies at a (111) interface in a CeO_2_–Y_2_O_3_ nanobrush, we performed a systematic STEM study. Figure 2 shows HAADF and low-angle annular dark-field (LAADF) images obtained from both the tip and middle parts of a [(CeO_2_)_6 u.c._/(Y_2_O_3_)_2 u.c._] nanobristle. In the HAADF images (Fig. 2a, b), the chevron pattern of a stack of alternating CeO_2_ and Y_2_O_3_ layers is clearly visible. As indicated in Fig. 2e, the angle between the two {111} facets is ~70.5°, which can readily be seen when viewed along the [110] direction, confirming the formation of (111) interfaces. The STEM images also reveal that the outer portion of each layer is thinner than its center part. In addition, a fast Fourier transform analysis clearly shows a fully coherent interface (i.e., free from any dislocations) formed between CeO_2_ and Y_2_O_3_ layers (Supplementary Fig. 6). Moreover, at the edge region of the bristles in the HAADF images, the CeO_2_ layers encase the Y_2_O_3_ layers. The capping yields vertically connected CeO_2_ (Supplementary Fig. 7). This may be attributed to the lower Ehrlich–Schwoebel barrier^29^ for CeO_2_ on Y_2_O_3_ than for Y_2_O_3_ on CeO_2_.

The two interfacial reconstruction scenarios mentioned previously, i.e., (1) formation of an oxygen interstitial on the Y_2_O_3_ side and (2) formation of an oxygen vacancy and subsequent Ce^3+^ ions on the CeO_2_ side, can result in a local lattice distortion. Such structural disorder can be detected by LAADF imaging, as it leads to electron dechanneling of the incident e-beam^30,31^. As shown in Fig. 2c, d, the LAADF images reveal an enhanced intensity at the interface between CeO_2_ and Y_2_O_3_. Through a careful examination of the images, we found that the bright contrast originated from the CeO_2_ side of the interfaces (see the yellow lines in Fig. 2a–d). Note that we have not observed any bixbyite Ce_2_O_3_ structures at the CeO_2_/Y_2_O_3_ interface, as confirmed by HAADF imaging (Supplementary Fig. 8). Considering the fact that the lattice mismatch between Ce_2_O_3_ and Y_2_O_3_ is ~10%, the formation of the bixbyite Ce_2_O_3_ phase may not be energetically favorable at the interfaces. LAADF imaging also confirmed that the structurally distorted layer owing to the chemical expansion spanned over ~1 nm in thickness (Supplementary Fig. 9). In addition, a previous TEM study reported a large lattice expansion occurred in CeO_2_ nanoparticles, owing to the formation of oxygen vacancies with Ce^3+^ ions, without changing the original fluorite structure^32^. Thus, our results suggest that the highly localized formation of a large number of oxygen vacancies on the CeO_2_ side of the interfaces was responsible for the observed electron dechanneling. The formation of interfacial oxygen vacancies was further confirmed by a geometric phase analysis (GPA) of STEM images. As shown in Fig. 2f, the GPA result reveals a significant lattice expansion by up to 3% for the CeO_2_ interfacial regions, consistent with the LAADF results. Furthermore, we can also exclude the possibility of the formation of oxygen interstitials on the Y_2_O_3_ side of the interface—one of the two interfacial reconstruction scenarios mentioned earlier based upon the STEM data—as we have not observed any atomic structural changes in Y_2_O_3_ interfacial layers.

### Interfacial cerium valence modulation

**Fig. 3:**
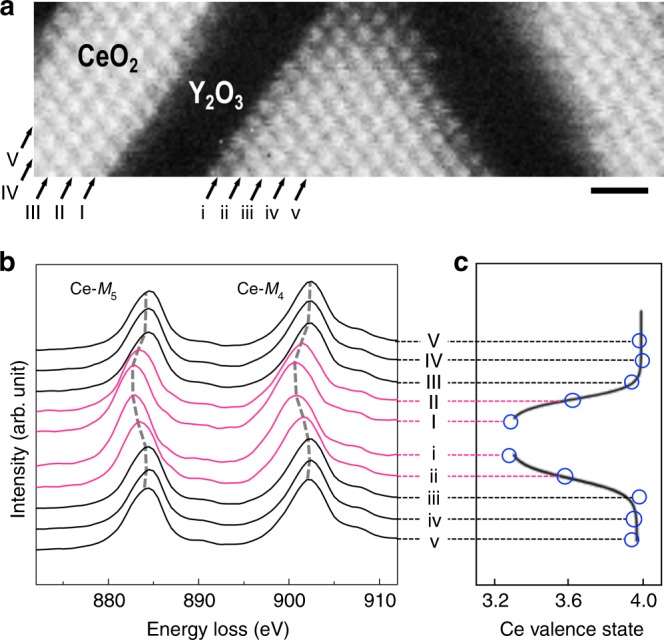
Interfacial valence changes seen by STEM/EELS. **a** Atomic-resolution EELS map for the Ce-*M*_4,5_ edges from a local area in a CeO_2_–Y_2_O_3_ nanobristle, revealing an atomically abrupt (111) interface. The scale bar corresponds to 1 nm. **b** Ce-*M*_4,5_ EELS spectra averaged along the (111)Ce atom planes. The dashed lines indicate the chemical shift of the Ce-*M*_5_ and Ce-*M*_4_ edges at the interface between CeO_2_ and Y_2_O_3_ as indicated by the EELS spectra in red. The latter were taken from the two interfacial CeO_2_ layers interleaved by a Y_2_O_3_ layer, indicating the presence of reduced CeO_2_ layers (~2 u.c. thick). **c** Average Ce valence states in the (111)Ce atom planes indicated in **b**, revealing the valence change in interfacial CeO_2_ layers.

The interfacial oxygen vacancy formation was further investigated by electron energy-loss spectroscopy (EELS), which highlighted the elemental distributions, atomic configurations, and bonding states of both Ce and O. Figure 3a shows an elemental map produced using the Ce-*M*_4,5_ edge in the vicinity of the (111)CeO_2_/(111)Y_2_O_3_ interface, revealing interfaces that are not only structurally well-defined but also chemically sharp. Background-subtracted Ce-*M*_4,5_ edge spectra obtained from each atomic plane across the (111)CeO_2_/(111)Y_2_O_3_ interface are shown in Fig. 3b. The peak positions of Ce-*M*_4,5_ edges are shifted to a lower energy-loss region for the reduced CeO_2_ interfacial layers, as compared with that of the CeO_2_ interior layer. The Ce-*M*_4,5_ edges originate from transitions between the 3*d* and 4*f* states (*M*_4_ corresponds to the 3*d*_3/2_ to 4*f*_5/2_ transition and *M*_5_ corresponds to the 3*d*_5/2_ to 4*f*_7/2_ transition) combined with ligand hole effects and valence-sensitive fine structures. Thus, spatially resolved chemical information^33,34^ was obtained from EELS measurements. Using linear combinations of Ce-*M*_4,5_ reference spectra from CeO_2_ and Ce_2_O_3_^34^, the valence states of the reduced cerium atoms near the interface were roughly estimated to be Ce^+3.3^ (the nearest to the interface) and Ce^+3.6^ (the second nearest), which correspond to CeO_1.65_ and CeO_1.8_, respectively, as illustrated in Fig. 3c. These oxidation states led to 17.5% and 10% of missing oxygen atoms, respectively, which was indeed a large amount, especially considering the robust interface lattice structures observed by STEM. Background-subtracted O-*K* spectra averaged along the (111)O planes show a considerable number of oxygen vacancies are formed at interfaces to cope with the interfacial charge mismatch (Supplementary Fig. 10). It is worth stressing that such an interfacial charge modulation is a powerful method of creating a high concentration of oxygen vacancies. Interestingly, our STEM and EELS results suggest that the formation of oxygen vacancies at the (111) interface between the fluorite and the bixbyite is not attributable to the creation of SCRs in the CeO_2_ layer, in which the calculated Debye length (λ) of ~2.4 nm at 300 K^16,35^ can result in a ~10 nm thick regime of overlapping space charges (4λ)^12^.

### Spatial distributions of oxygen defects at the (111) interface

**Fig. 4:**
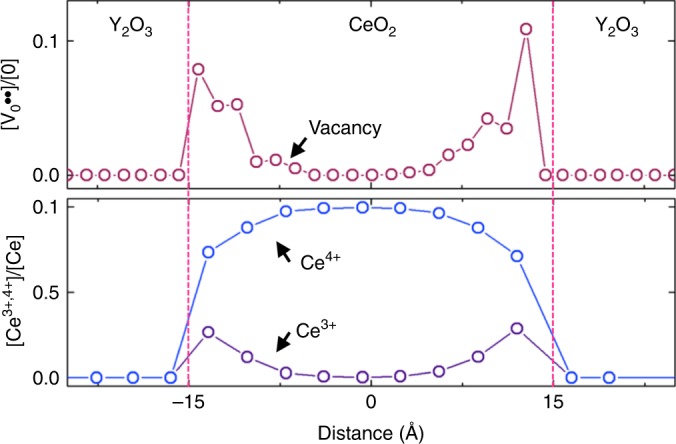
Cation and oxygen vacancy distributions at the (111) CeO_2_/Y_2_O_3_ interface. Distribution profiles of oxygen vacancy (top) and Ce^3+^, Ce^4+^ (bottom) equilibrated at 700 °C in CeO_2_ and Y_2_O_3_ calculated by hybrid Monte Carlo (MC) and molecular dynamics (MD) simulations. The interfaces enrich Ce^3+^ and oxygen vacancies simultaneously within CeO_2_. The concentration of oxygen vacancies can reach up to 10% at the interface, spanning over ~1 nm.

To clarify the role of the (111) interface in creating the oxygen vacancies, the defect formation energy of a neutral oxygen vacancy complex, $({\mathrm{Ce}}_{{\mathrm{Ce}}}^\prime - {\mathrm{V}}_{\mathrm{O}}^{ \cdot \cdot } - {\mathrm{Ce}}_{{\mathrm{Ce}}}^\prime )^ \times$, at the (111) interface was computed by density functional theory (DFT) calculations (Supplementary Fig. 11). The DFT calculations indicated that the positive charge of oxygen vacancies was always compensated by two Ce^3+^ ions (Supplementary Fig. 12). In addition, the DFT results revealed that the formation energies of oxygen vacancies and Ce^3+^ ions at the heterointerface were ~1.8 and ~0.5 eV, respectively. These values are lower than those in bulk CeO_2_ as a result of the lower electronic state of the polaron (i.e., reduced cerium cations, ${\mathrm{Ce}}_{{\mathrm{Ce}}}^\prime$) and the easier local lattice relaxation at the interface. These low formation energies increased the number of both the oxygen vacancies and the Ce^3+^ ions at the interface, leading to the segregation of oxygen vacancies on the CeO_2_ side of the interface, as shown in Fig. 4. The calculation result showing up to 10% oxygen loss at the interface is quantitatively in good agreement with experimental observations of the EELS data shown in Fig. 3b, c. It is also worth noting that the vacancy segregation was not driven solely by an electrostatic potential at the interface, as the (111) surface was nonpolar so that an SCR could not easily form, and the defect concentration in ceria was high enough to screen out any electrostatic potential (Supplementary Fig. 13). Therefore, the (111) fluorite–bixbyite interface could induce a larger number of oxygen vacancies without the creation of an SCR, which differs from the mechanism proposed for (001) interfaces of fluorite–bixbyite systems in previous studies^15,16^. As was confirmed by STEM imaging, classical force field (FF) simulation also confirmed that the CeO_2_ side of the interfaces always remained as the fluorite structure, even though the interface had a high concentration of oxygen vacancies (Supplementary Fig. 14).

## Discussion

There have been several studies of creating oxygen defects, understanding the underlying mechanism for their formation, and probing their existence in mixed ionic and electronic conducting materials^13,14^. However, identifying a new means to artificially create and control highly confined interfacial oxygen vacancies has been a huge challenge. The present work elucidates that interfaces between the fluorite CeO_2_ and the bixbyite Y_2_O_3_ can provide an excellent platform for developing new ionic materials. Creating atomically sharp (111) fluorite/bixbyite interfaces in the nanobrush architecture provides a completely new approach to spontaneously creating oxygen vacancies through interfacial charge modulation. In contrast to the well-known SCR effect, the (111) fluorite/bixbyite interface creates and confines oxygen vacancies within two to three atomic layers on the CeO_2_ side of the (111) interface. It is remarkable that a large number of oxygen vacancies can be accommodated by taking advantage of the novel interface architecture while leaving the lattice structure intact.

We further demonstrated a new “synthesis science” approach^36^ to making a specific interfacial nanomaterial with a vertically aligned geometry and a largely increased area of the (111)-oriented surface. As shown in Fig. 1a, our CeO_2_–Y_2_O_3_ nanobrush has a highly porous topography. Therefore, the quantitative information on porosity was important for accurately determining the surface area, which was an extremely challenging task. The difficulty was overcome by using small-angle neutron scattering (SANS), as shown in Supplementary Fig. 15. The SANS results revealed that the upper limit of the porosity was ~49%. More details on the SANS measurement and data analysis can be found elsewhere^28^. The high porosity is highly advantageous for many technical applications, in which a large contact area can improve (electro-)chemical activity, e.g., electrodes of solid oxide fuel/electrolysis cells^37^, oxygen permeation membranes^38^, chemical sensors^39^, and catalysts^40^. In our study, the SANS data confirmed that the ~1.4-μm-thick nanobrushes had a surface area that is over 200 times greater than that of a 2D thin film. Therefore, these advances are major technical accomplishments that provide a new means to develop high-performance energy conversion devices, novel catalysts, nonvolatile memories, and memristive computing devices.

## Methods

### Synthesis of nanobrush samples

Micron-thick single-crystalline fluorite–bixbyite (CeO_2_–Y_2_O_3_) nanobrush superlattices were fabricated using pulsed laser epitaxy (KrF, *λ* = 248 nm) with sintered CeO_2_ and Y_2_O_3_ targets. While all of the samples studied were grown on single-crystal (001) YSZ substrates, the samples for STEM and APT were grown on YSZ-buffered (001) silicon substrates without deteriorating the sample quality to avoid the charging effect resulting from the highly insulating YSZ substrate. Details on the growth of a YSZ buffer layer and the epitaxial relationship of YSZ and silicon can be found elsewhere^41–44^. All of the samples were deposited at 700 °C at an oxygen partial pressure of 100 mTorr. The sample structure and crystallinity were characterized by high-resolution four-circle XRD.

### Scanning transmission electron microscopy

Cross-sectional TEM specimens were prepared using ion milling after mechanical thinning and precision polishing. HAADF and LAADF imaging and EELS analysis were carried out in a scanning transmission electron microscope (Nion UltraSTEM100) operated at 100 keV. The microscope is equipped with a cold field-emission gun and an aberration corrector for sub-Ångstrom resolution. Inner detector angles of 78 and 30 mrad were used for HAADF and LAADF imaging, respectively. The convergence semiangle for the electron probe was set to 30 mrad.

### Atom probe tomography

APT specimens were prepared by first coating a silicon microtip array with the CeO_2_/Y_2_O_3_ nanobristles. An FEI Nova 200 dual-beam scanning electron microscope/FIB was used to fabricate APT needles from the nanobrushes grown on the silicon microtip array. A 300 nm platinum layer was deposited by FIB, followed by several annular milling steps and a final 2 kV mill to fabricate needle-shaped specimens ready for APT analysis. After sample preparation, the specimen was loaded into the LEAP 4000XR and analyzed using laser mode with a 10 ps/100 pJ laser pulse, a 50 K base temperature, a 125 kHz pulse repetition rate, and a detection rate of 1 ion per 500 pulses. Reconstruction and analysis were performed using the CAMECA integrated visualization and analysis software package.

### Density functional theory calculations

In this work, the DFT calculations were performed with VASP^45–47^ using the projector augmented wave method. Generalized gradient approximation with the PBE exchange correlation functional^48^ and an energy cutoff of 500 eV were used. To properly simulate Ce^3+^, a Hubbard U correction^49^ was applied to the Ce 4*f* orbital with *U* = 5 eV. The model of the CeO_2_/Y_2_O_3_ {111} interface was constructed according to the TEM observation, in which Y_2_O_3_ was stretched biaxially by 1.4% and CeO_2_ was compressed by 1.4%. The interface model consisted of 24 Y_2_O_3_ and 48 CeO_2_. The simulation cell was hexagonal with *a* = 1.535 nm, *c* = 1.880 nm, and *γ* = 120°. Both the CeO_2_ layer and the Y_2_O_3_ layer were 0.94 nm thick. For all calculations, a *k*-point mesh of 2 × 2 × 1 was used. The vacancy formation energy was computed by removing a neutral oxygen atom close to the interface or in the middle of the CeO_2_ layer.

### Hybrid Monte Carlo and molecular dynamics with classical force field

The vacancy and Ce^3+^ concentration profiles were computed with a hybrid MC and MD algorithm using a classical FF. The simulation technique is well documented in the original paper^50^ and our previous work^51^. The simulation was started with a uniform distribution of Ce^3+^ and oxygen vacancies in the CeO_2_ layer. In the MC simulation, only Ce^3+^ and Ce^4+^ ions were allowed to swap their positions, whereas in the MD simulation, the oxygen vacancies could diffuse to either Y_2_O_3_ or CeO_2_ layers. More than 10^5^ MC trials and 6 ns MD simulations at 600 °C were performed. The defect concentration profiles were obtained by averaging the last 5000 configurations of each single calculation.

### Small-angle neutron scattering

Room-temperature SANS measurements were performed using EQ-SANS at the Spallation Neutron Source at Oak Ridge National Laboratory^52,53^. Data were collected in 60 Hz mode with a *Q*-range between ~0.015 and 0.35 Å^−1^. The 2D scattering data were reduced using the MantidPlot software according to standard procedures^54^. The SANS signals from CeO_2_–Y_2_O_3_ nanobrush samples were subtracted the background scattering from a YSZ bare substrate.

## Supplementary information

Supplementary Information

Supplementary Movie 1

## Data Availability

Data supporting the findings of this study are available from the corresponding author upon reasonable request.

## Source data

Source Data

## Code availability

The VASP code, which is available for purchase, was used for the calculations in this paper. The configuration files used in these calculations are all available upon request from the authors.
