# Supplementary material for: Colossal oxygen vacancy formation at a fluorite-bixbyite interface
Source: Nat Commun. 2020 Mar 13;11:1371. doi: 10.1038/s41467-020-15153-8 (PMC7069997; doi:10.1038/s41467-020-15153-8)
Supplement: Supplementary file 1 — Supplementary Information [file 41467_2020_15153_MOESM1_ESM.pdf]

Supplementary Information for

Colossal oxygen vacancy formation at a fluorite-bixbyite interface

Lee et al.

This PDF file includes:

Supplementary Figs. 1 to 15

Other Supplementary Information for this manuscript include the following:

Supplementary Movie 1 (.mp4)

Supplementary Movie 1 | Three-dimensional geometry of free-standing CeO_2-Y_2O_3 nanobrush superlattices from atom probe tomography (APT). An atom probe tomography reconstruction with Ce and Y atoms displayed as red and blue points, which shows a three-dimensional view of the internal structure of free-standing CeO_2-Y_2O_3 nanobrush superlattices.

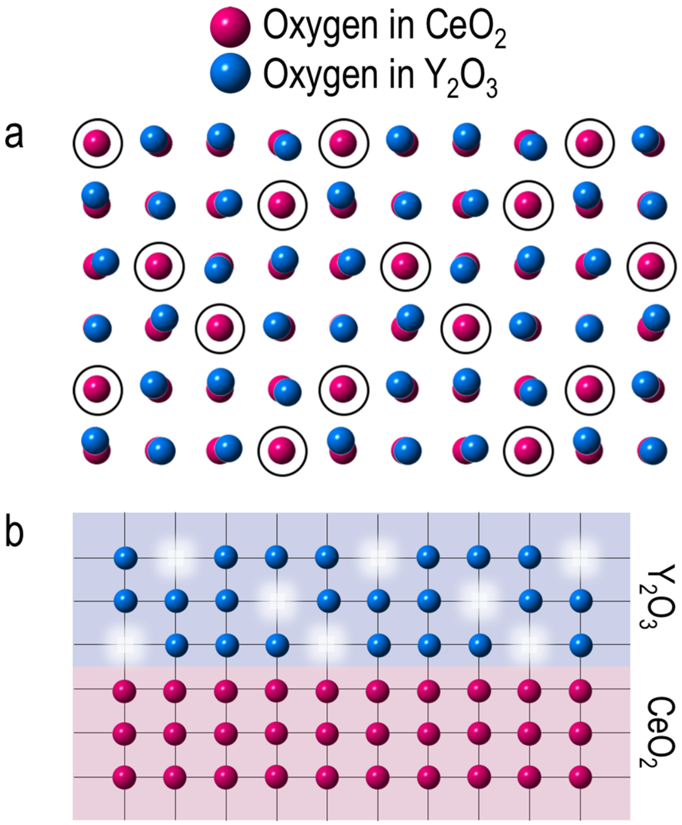

Supplementary Fig. 1. | Schematic illustration of anion arrangement at a (001) interface between fluorite CeO_2 and bixbyite Y_2O_3. a Top and **b** cross-sectional views of a (001) interface between CeO_2 and Y_2O_3. Cations are not shown. Red and blue spheres represent oxygen in CeO_2 and Y_2O_3, respectively. Black circles indicate excess oxygen atoms in CeO_2.

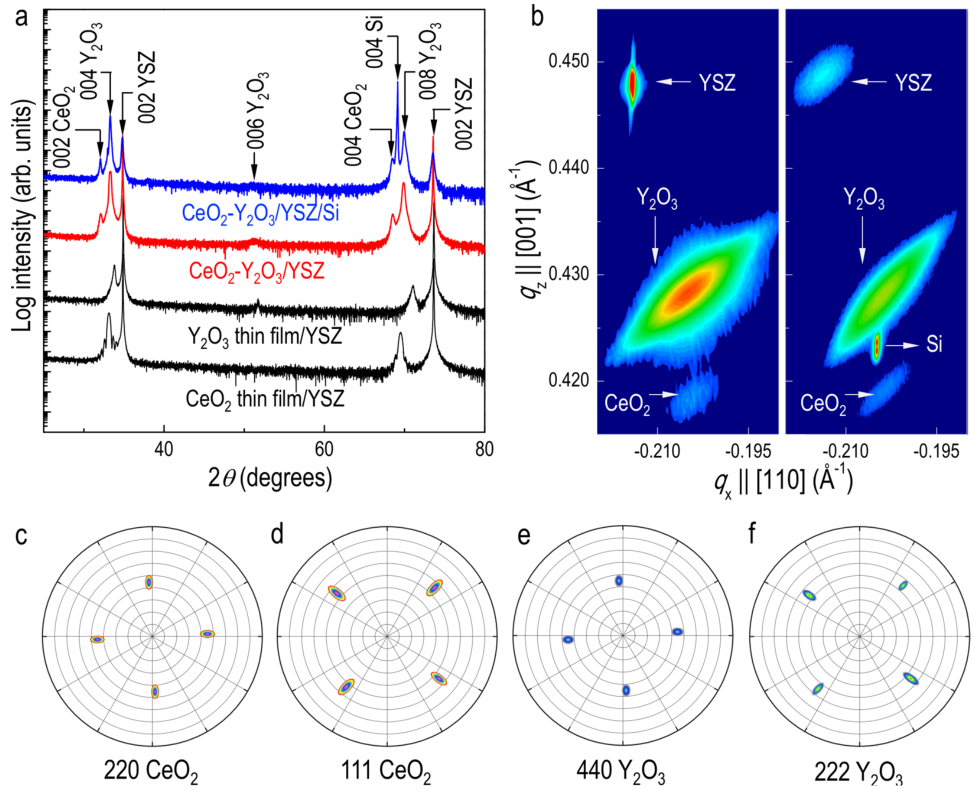

Supplementary Fig. 2. | Epitaxial synthesis of free-standing CeO_2-Y_2O_3 nanobrush superlattices. a XRD θ-2θ diffraction patterns of the free-standing CeO_2-Y_2O_3 nanobrush superlattices on (001) Si with a YSZ buffer layer (blue) and on (001) YSZ (red). XRD θ–2θ diffraction patterns for epitaxial CeO_2 and Y_2O_3 films (black) on (001) YSZ are also shown. **b** X-ray reciprocal space maps (RSMs) around the -1-13 Bragg peak from the free-standing CeO_2-Y_2O_3 nanobrush superlattices on (001) YSZ (left) and on (001) Si with a YSZ buffer layer (right). Both XRD θ-2θ diffraction patterns and X-ray RSMs indicate that the crystallography of free-standing CeO_2-Y_2O_3 nanobrush superlattices retains regardless of the substrate. **c-f** X-ray pole figures of the free-standing CeO_2-Y_2O_3 nanobrush superlattices on (001) YSZ. The pole figures were plotted with the pole distance angle ψ=0° (center) to ψ=90° (rim). Four diffraction peaks with a fourfold symmetry are seen at ψ=45° showing that CeO_2 {220} planes (**c**) and Y_2O_3 {440} planes (**e**) have a tilt angle of 45° with respect to the (002) YSZ. In (**d**) and (**f**), CeO_2 {111} planes (**d**) and Y_2O_3 {222} planes (**f**) have a tilt angle of 54.7° with respect to the (002) YSZ as four diffraction peaks with a fourfold symmetry are also seen.

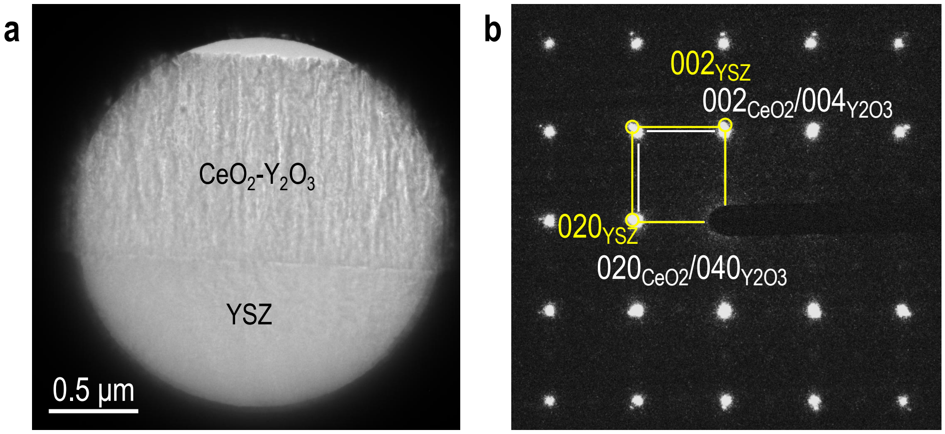

Supplementary Fig. 3. | Epitaxial relationship of a free-standing CeO_2-Y_2O_3 nanobrush superlattice and substrate. **a** Bright-field TEM image showing the selected area covering nanobrush/substrate. **b** Selected area electron diffraction obtained along the [100]CeO_2-Y_2O_3 zone-axis. The result reveals a crystal orientation of (001)CeO_2-Y_2O_3/(001)YSZ between the nanobrush and substrate.

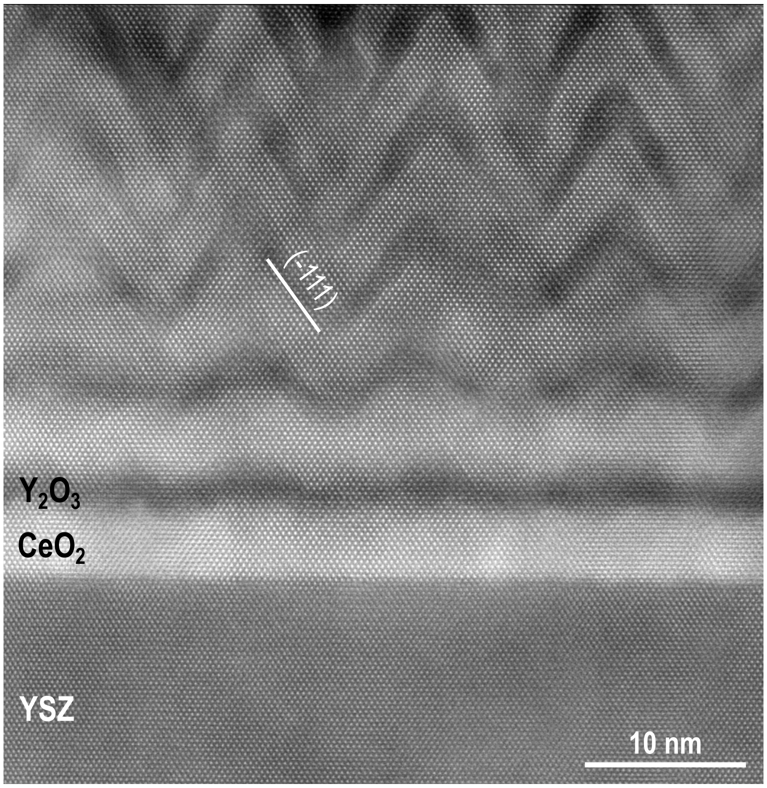

Supplementary Fig. 4. | Growth mechanism of a free-standing CeO_2-Y_2O_3 nanobrush superlattice. At the initial stage of the growth, a 3D island-like morphology with {111} facets is formed as a result of both the suppressed surface diffusion and the high thermodynamic stability of the {111} facets. After the initial stage, the growth transition into the individual columnar structure occurs as a result of diffusion-limited aggregation (DLA) associated with the fast growth that limits surface diffusion and the shadow effect. Finally, micron-thick, porous oxide nanosuperlattices with the facets of {111} planes are formed.

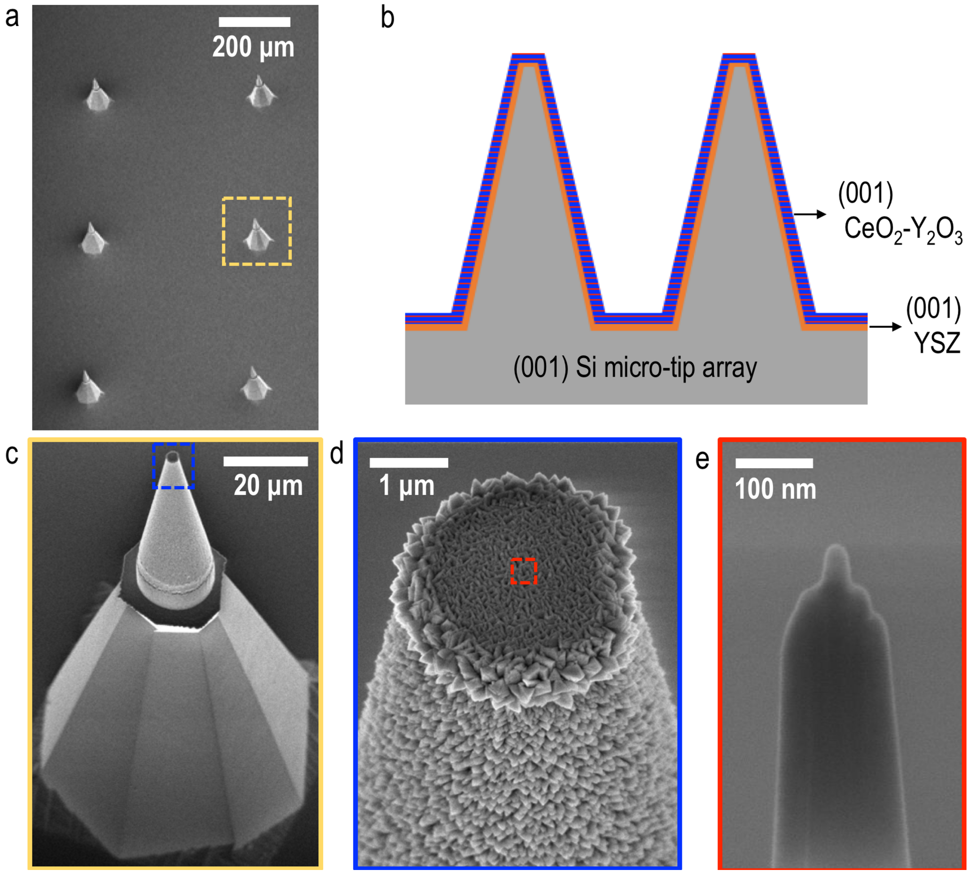

Supplementary Fig. 5. | Sample preparation of free-standing CeO_2-Y_2O_3 nanobrush superlattices grown on a (001) Si micro-tip array with a buffered YSZ for atom probe tomography (APT). **a** A low magnification scanning electron microscopy image of CeO_2-Y_2O_3 nanobrush superlattices deposited on a (001) Si micro-tip array with a YSZ buffer layer. **b** A schematic of the YSZ and CeO_2/Y_2O_3 layers grown on the Si micropost array. **c** A magnified SEM image of the yellow box region in (**a**). **d** A magnified SEM image of the blue box region in (**c**). **e** An SEM image of an isolated CeO_2/Y_2O_3 nanobrush after final FIB milling ready for APT analysis.

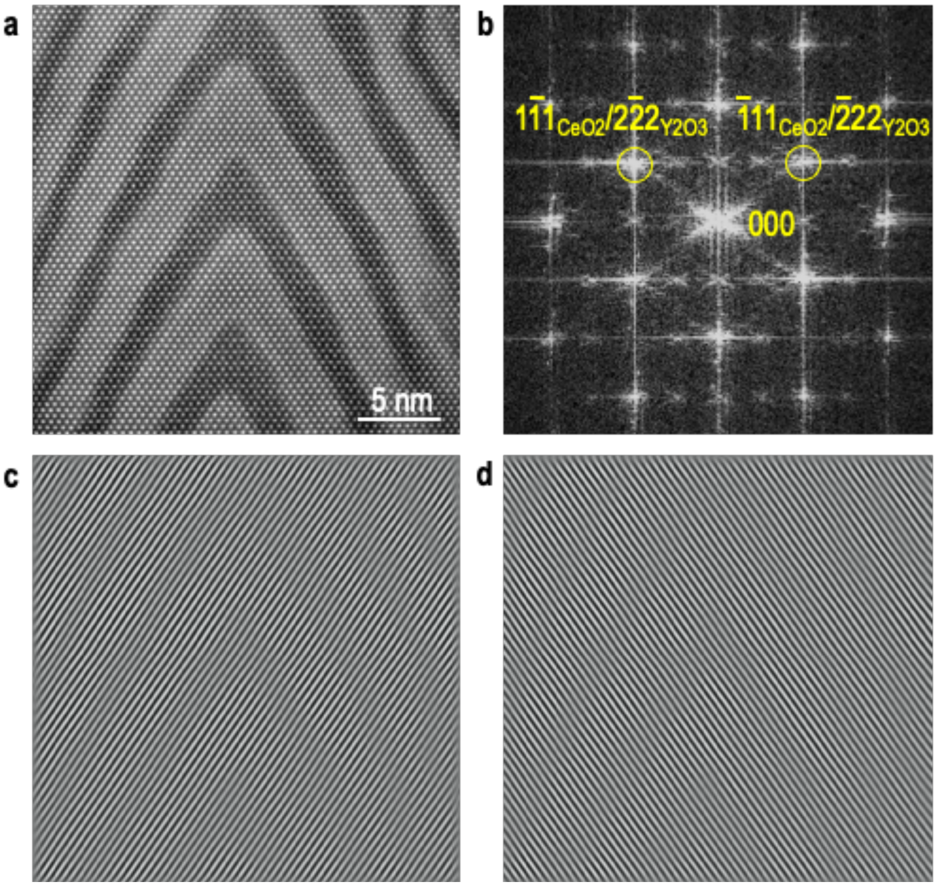

Supplementary Fig. 6. | Coherent interface structure of a CeO_2/Y_2O_3 nanobrush superlattice. **a** HAADF image showing the atom structure projected along the [110] zone-axis. **b** Corresponding fast-Fourier transform (FFT) pattern of (a). **c**, **d** An inverse FFT image from the FFT pattern in (a) using the (c) $(1\bar{1}1)CeO_2/(2\bar{2}2)Y_2O_3$ and (d) $(\bar{1}11)CeO_2/(\bar{2}22)Y_2O_3$ pairs. The result clearly shows a fully coherent interface (i.e., free from any dislocations) formed between the CeO_2 and Y_2O_3 component layers.

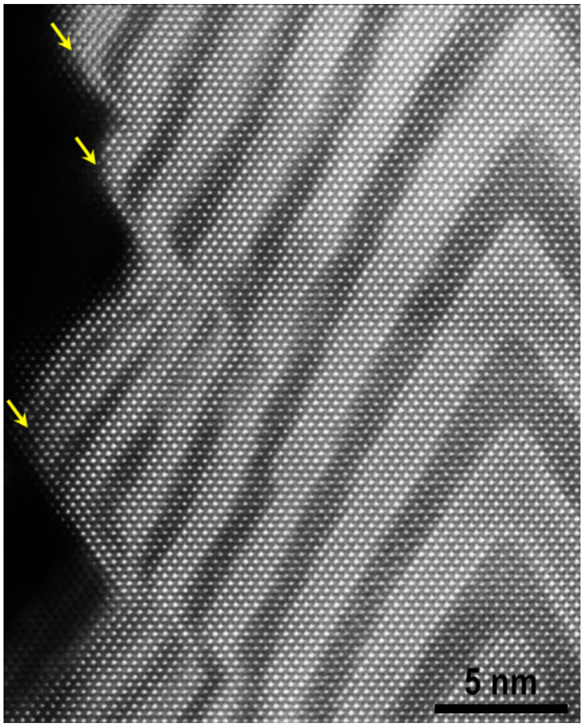

Supplementary Fig. 7. | Atom distributions at the surface of free-standing CeO_2/Y_2O_3 nanobrush superlattices. The cation atoms at the surface facets (as arrowed, 2-3 atom planes) show uniformly and clearly a brighter contrast than the Y atoms in the HAADF image, which was taken along the [110] direction of the nanobrush superlattice. The result suggests that the nanobrush superlattice surfaces are fully coated by a thin CeO_2 layer, which connects all the inner V-shaped CeO_2 layers to form a highly conductive network.

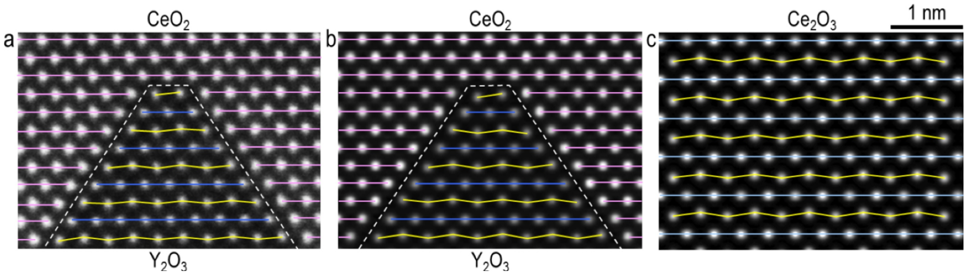

Supplementary Fig. 8. | Atom arrangements in the interface layer. a Experimental HAADF image taken from a CeO_2-Y_2O_3 nanobrush superlattice along its [110] direction. **b** Simulated HAADF image of (**a**). In (**a**) and (**b**), the interface between CeO_2 (top) and Y_2O_3 (bottom) layers is indicated by a dashed line. **c** Simulated HAADF image of Ce_2O_3 along its [110] direction. Crystal structures of stoichiometric CeO_2, Y_2O_3 and Ce_2O_3 phases were used for image simulation. Yellow lines in all the figures indicate the zig-zag atom column displacements in every another (001)Y or (001)Ce atom planes, while the straight lines indicate the rest of the (001)Y or (001)Ce atom planes where the atom displacement is essentially absent. The zig-zag atom column displacement, which features the bixbyite structure of Y_2O_3 and Ce_2O_3 was not observed in the entire CeO_2 layer. The excellent match between the experimental and simulated HAADF images suggests a uniform atom configuration throughout the CeO_2 layer (including the interfacial layer).

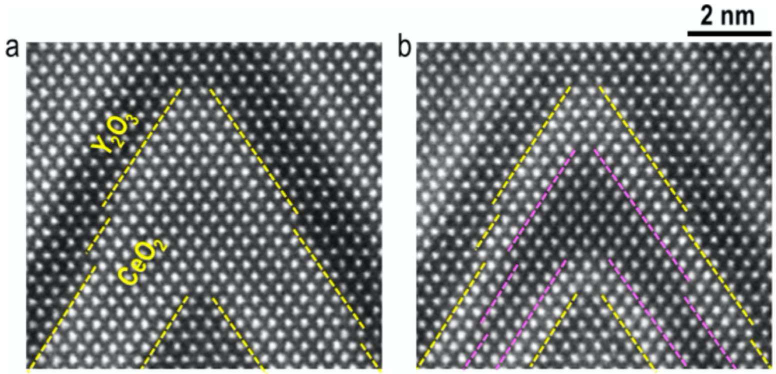

Supplementary Fig. 9. | Thickness of the interface layer. a HAADF and **b** LAADF images taken from the same region of free-standing CeO_2-Y_2O_3 nanobrush superlattices along its [110] direction. The yellow dashed lines indicate the outmost {111} facets of the CeO_2 layer in both images, and the magenta dashed lines indicate the inner {111} facets of the brighter interfacial layer in LAADF images. A thickness of 2-3 Ce atom planes of the interfacial layer is obvious.

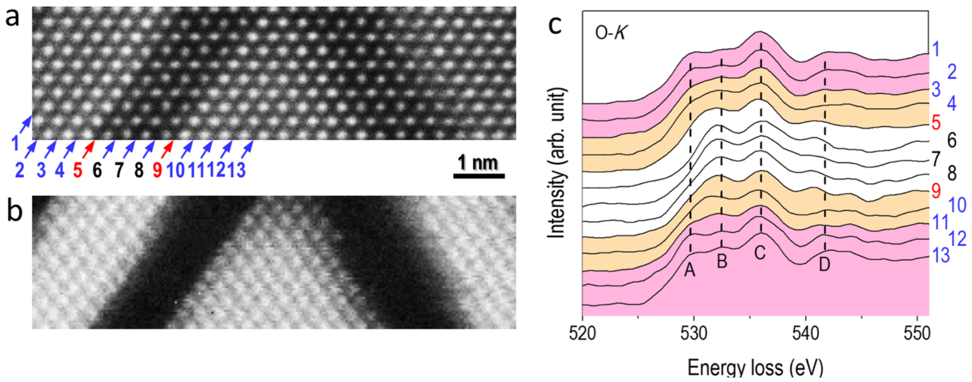

Supplementary Fig. 10. | Atom configurations and bonding states from EELS. a HAADF image of CeO_2/Y_2O_3 interface along its [110] direction. **b** Atomic-resolution Ce-$M_{4,5}$ edge map obtained from the local area shown in (**a**). Both (**a**) and (**b**) reveal not only structurally well defined, but also chemically sharp interfaces. **c** Background-subtracted O-*K* spectra averaged along the (111)O planes, as labeled in (**a**). The dashed lines marked in (**c**) indicate the chemical shift the O-*K* white lines of the outer layer Ce atoms relative to that of the internal Ce atoms. The O–*K* edge spectra for the two outermost layers interfacing with Y_2O_3 (labeled #3, 4, 10, and 11) show significant decreases in the intensity of peak A as compared to the interior layers of CeO_2 (#1, 2, 12, and 13). The suppression of the O–*K* edge peak A indicates that the outer layers contain a considerable amount of oxygen vacancies formed to cope with the interfacial charge mismatch. In addition to the reduced intensity, the O–*K* edge spectral features from the interfacial layers in CeO_2 are similar to those obtained from the Y_2O_3 layers. The spectral shape changes occurred in the two outermost interfacial layers. This two layer thickness, where the spectral shape changes, is consistent with that revealed by the LAADF imaging. Thus, we conclude that the bright contrast in the LAADF images was caused by oxygen vacancies formed in CeO_2. The EEL spectra from the interfacial oxygen layers connecting the neighboring Y and Ce atom planes unambiguously show the valence reduction of Ce. The spectral shape from the interfacial CeO_2 layers is more or less similar as for Y_2O_3. However, we note that the reduced $CeO_{2-\delta}$ interfacial layers still maintain the original fluorite structure, which can also be supported by the fact that the fluorite structure has no such zig-zag positioning of the cation (Supplementary Fig. 8) while the bixbyite structure shows the characteristic alternative zig-zag pattern coming from the six-fold oxygen coordination.

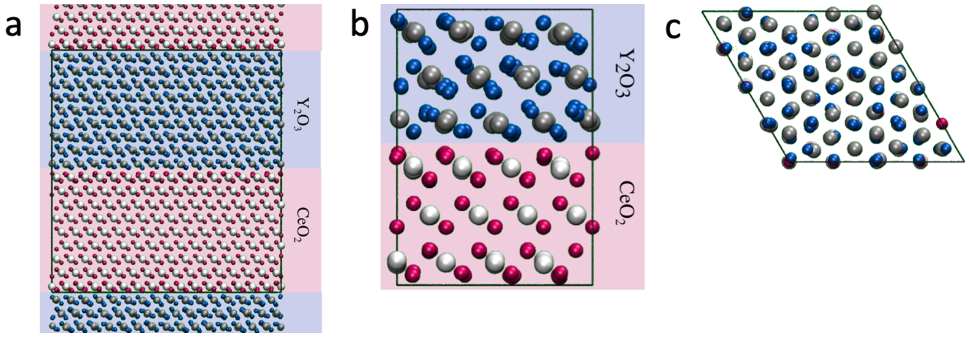

Supplementary Fig. 11. | Interface models for **a** classical force field simulations. The cell size is 3×5.3×5.6 nm^3 with 2.8 nm thickness for each layer. The cell contains 6336 atoms. **b** density functional simulation. The cell size is 1.5×1.5×1.9 nm^3 with 264 atoms. **c** The top view of the DFT simulation cell. White, grey spheres represent Ce and Y ions respectively, while both blue and red spheres represent O ions.

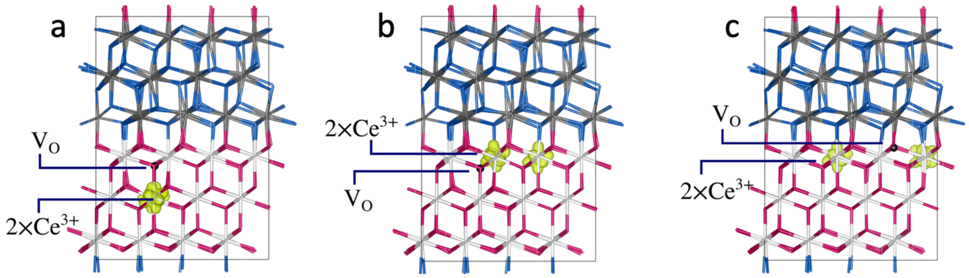

Supplementary Fig. 12. | Neutral vacancy configurations. a Ce^{3+} and V_O are all away from the interface, $E_f = 3.73$ eV. **b** The two Ce^{3+} are close to the interface, $E_f = 3.24$ eV. **c** Ce^{3+} and V_O are all close to the interface. $E_f = 1.94$ eV. White, grey spheres represent Ce and Y ions respectively, while both blue and red spheres represent O ions. The vacant sites are marked by black spheres. The local spin density is depicted by yellow isosurfaces.

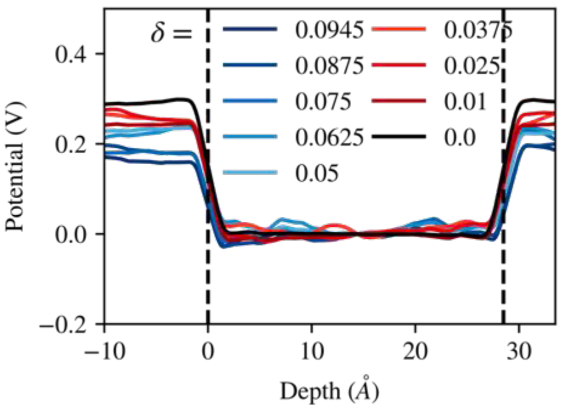

Supplementary Fig. 13. | Interface potential profile for δ = 0 ~ 0.0945. The Y_2O_3 part always have a higher potential than the CeO_2 part. This is because the bixbyite structure has less O^{2-} and a lower oxidation state of the cation, which leads to a higher bulk Madelung potential is Y_2O_3. This potential difference decreases at a larger δ, since more cations in the CeO_2 part is 3+ oxidation state and there are less O^{2-} ions. The strong Ce^{3+}-V_o attraction significantly reduces any possible impact from the space charge. That is why the electrostatic potential is flat at both sides of the interface and the electric field only exists in a 0.2 nm thick region at the interface.

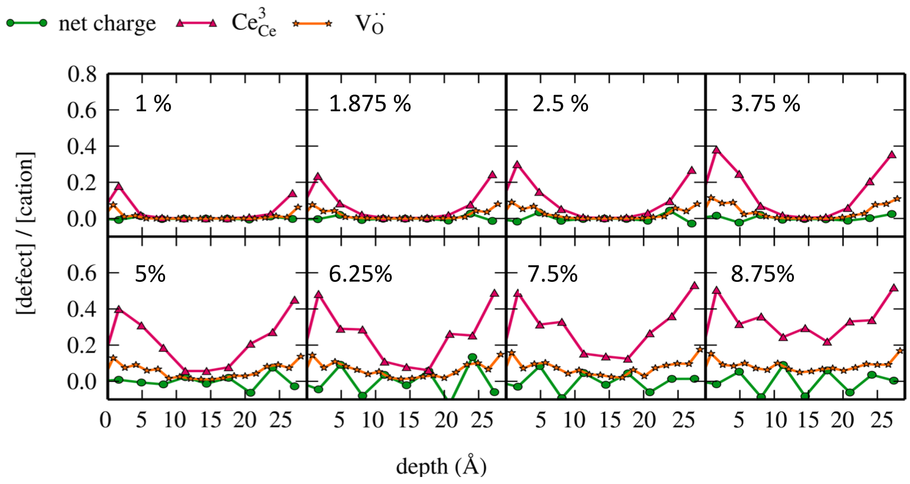

Supplementary Fig. 14. | Equilibrium distribution of Ce^{3+} , oxygen vacancy and net charge. Both ends of each plot are the interfaces between Y_2O_3 and CeO_2. Only the CeO_2 side is plotted. The average Ce^{3+} concentration is labeled in the plot. The δ for each case is labeled in the plot. The highest local Ce^{3+} concentration at the interface is found to be 45%, where the oxygen vacancy concentration at the interface is 16%. Considering that a bixbyite phase contains 100% Ce^{3+} with 25% of oxygen vacancies, the CeO_2 in the nanobrush always remains as the fluorite structure.

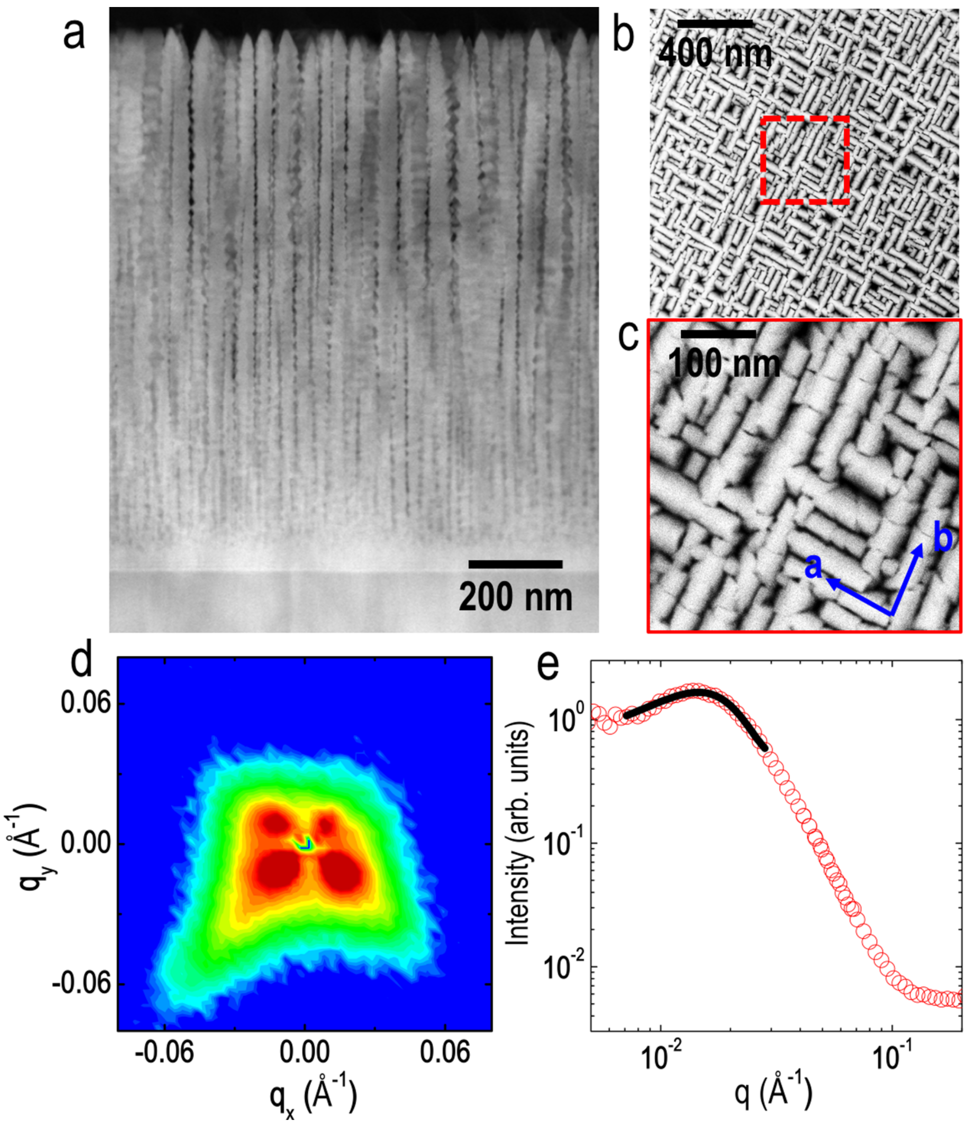

Supplementary Fig. 15. | Topography and porosity of free-standing CeO_2-Y_2O_3 nanobrush superlattices. a-c HAADF-STEM images of free-standing CeO_2/Y_2O_3 nanobrush superlattices grown on a (001) YSZ-buffered Si substrate, taken along the $[110]_{YSZ}$ direction. **a** Cross-sectional HAADF-STEM image of free-standing CeO_2/Y_2O_3 nanobrush superlattices. **b** Low-magnification plan-view HAADF images of free-standing CeO_2/Y_2O_3 nanobrush superlattices and **c** a magnified image of the region indicated by a red dashed square in (**b**). **d-e** Accurate determination of porosity determined by neutron measurements. **d** EQ-SANS data for free-standing CeO_2-Y_2O_3 nanobrush superlattices and **e** integrated radial intensity from (**d**). Solid line (black) indicates the Gaussian fit to the peak of the radial average.
